# Supplementary material for: In situ cryo-ET visualization of mitochondrial depolarization and mitophagic engulfment
Source: Proc Natl Acad Sci U S A. 2025 Jul 31;122(31):e2511890122. doi: 10.1073/pnas.2511890122 (PMC12337332; doi:10.1073/pnas.2511890122)
Supplement: Supplementary file 1 — Appendix 01 (PDF) [file pnas.2511890122.sapp.pdf]

Supporting Information for

In situ cryo-ET visualization of mitochondrial depolarization and mitophagic engulfment

Kevin Rose, Eric Herrmann, Eve Kakudji, Javier Lizarrondo, A. Yasemin Celebi, Florian Wilfling, Samantha C. Lewis, and James H. Hurley

James H Hurley

Email: jimhurley@berkeley.edu

This PDF file includes:

Figures S1 to S5

Legends for Movies S1-S5

Other supporting materials for this manuscript include the following:

Movies S1-S5

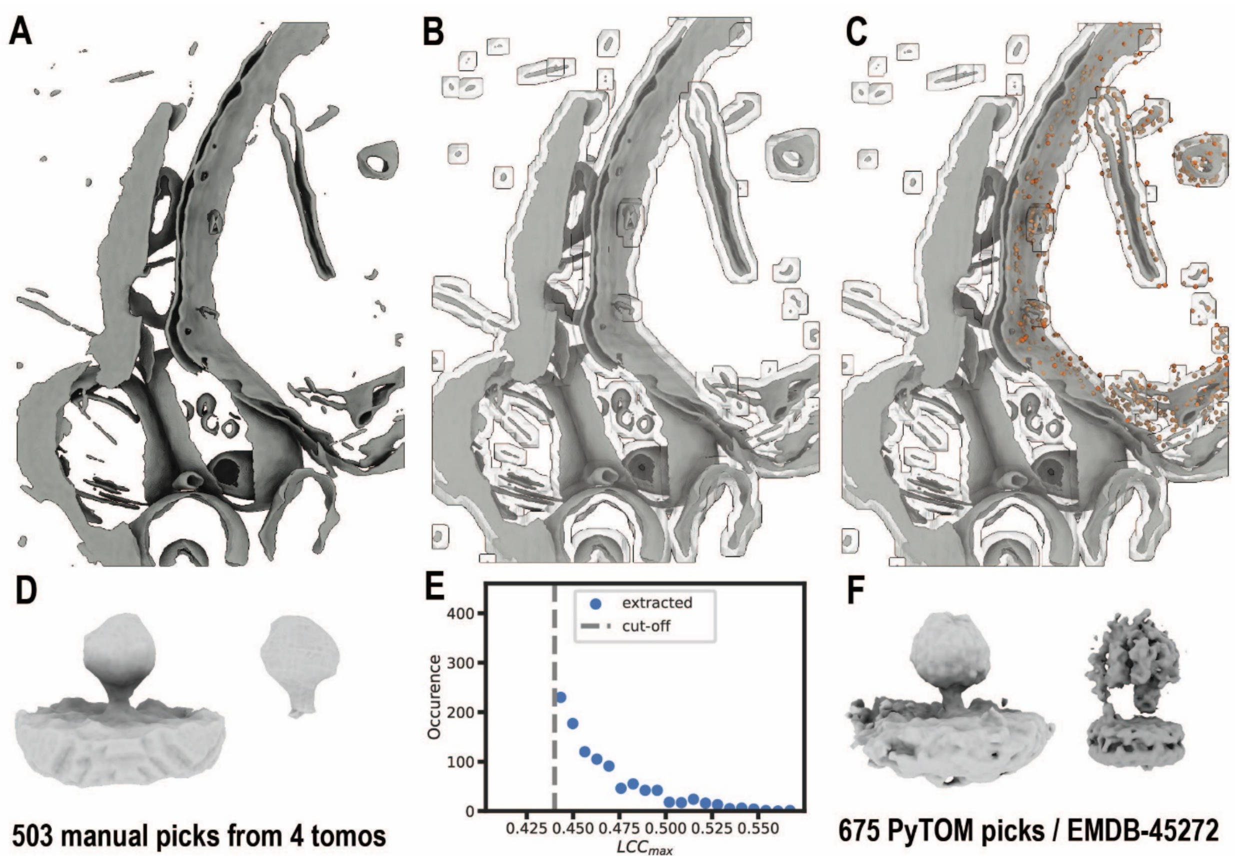

Supplemental Figure 1: Template match picking strategy for ATP synthase. Membranes from tomograms were first segmented to guide the particle picking process (A). A mask to guide template match picking was generated using the membrane segmentation (B). Output of template match picking (C) using the resulting sub-tomogram average from 503 manual picks of 4 tomograms with membrane (left) or without (right) (D). (E) PyTOM parameters used to restrain template matching. Initial sub-tomogram average from 675 template match picks from PyTOM compared to a recently solved density map (EMDB-45272) (F).

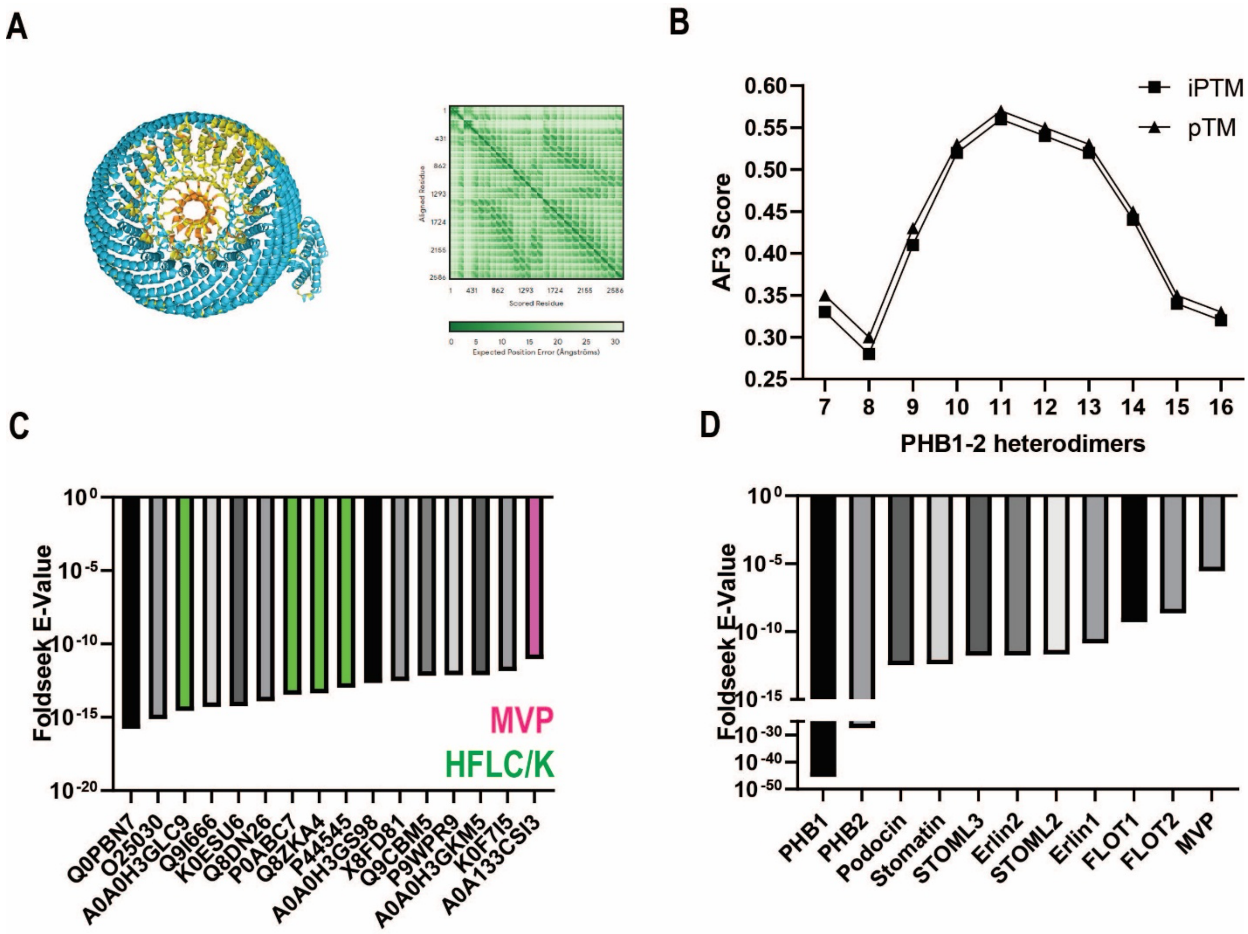

Supplementary Figure 2: Structural analysis and comparison of Prohibitin and the closely related HFLK/C complexes. Alphafold modeling of a heterododecamer Prohibitin 1-2 complex and resultant pLDDT plot from Alphafold 3 with 2 heterodimer copies (A). Alphafold 3 screen using 2 heterodimer copies shows a preferred stoichiometry between 11 and 12 heterodimer copies (B). Foldseek using the structure of human prohibitin shows greater E-Values to bacterial HFLC/K structures than to other closely related mammalian SPFH domain-containing proteins (C-D).

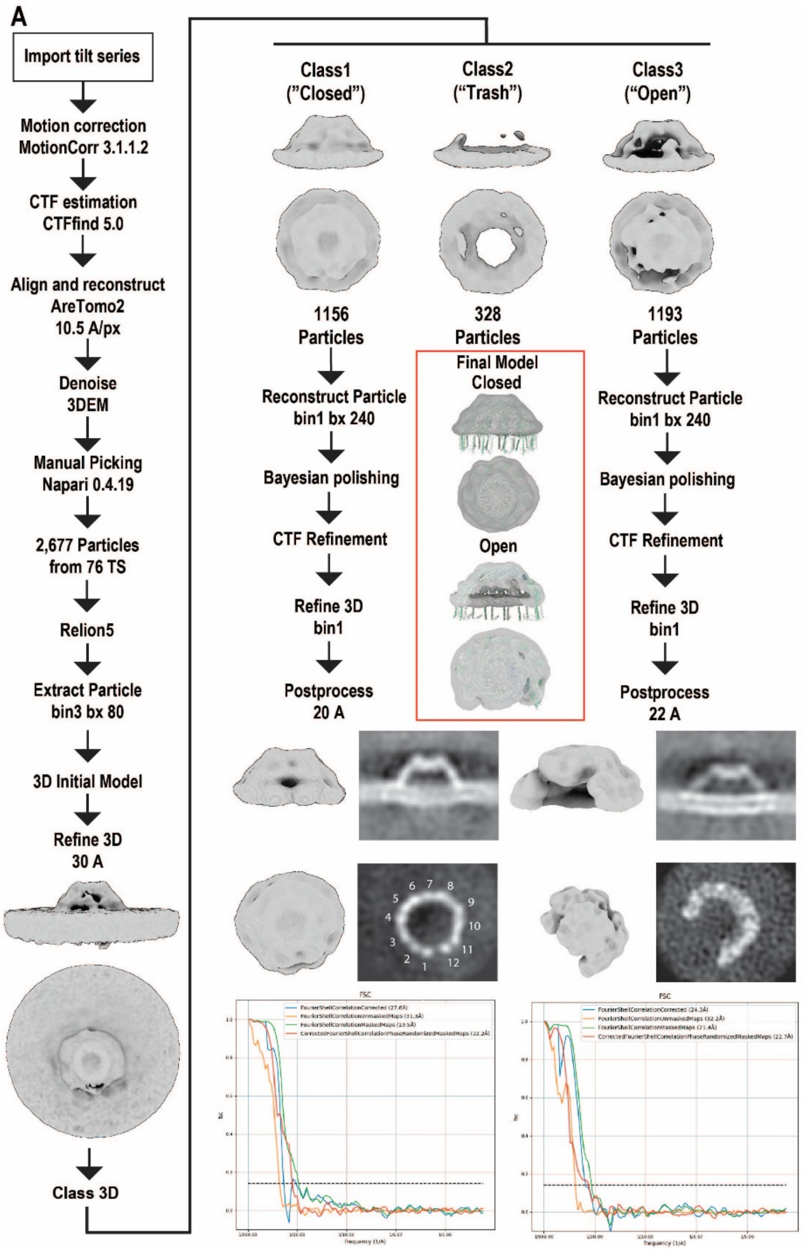

Supplementary Figure 3: Data processing pipeline for sub-tomogram averaging of the prohibitin complex.

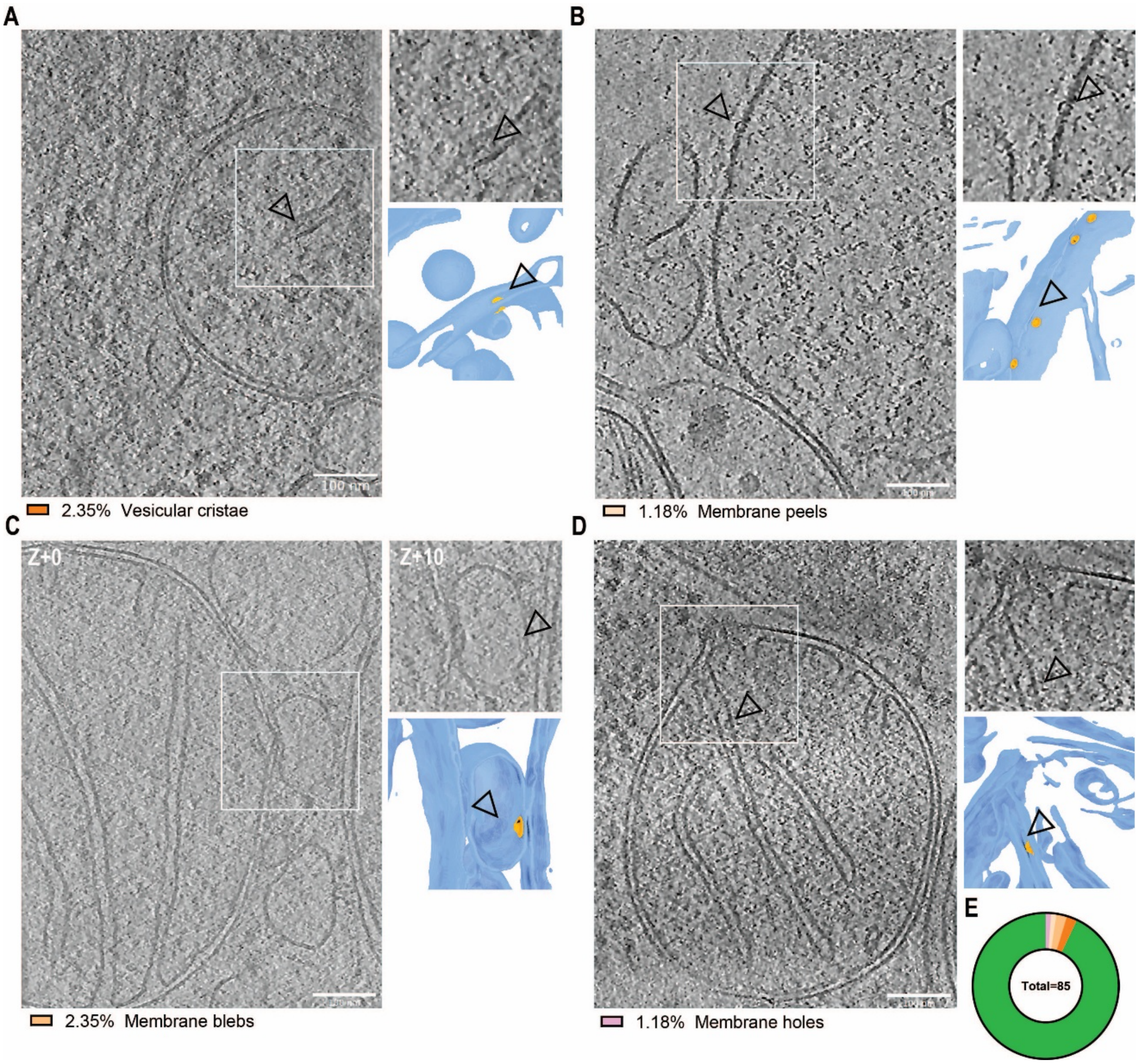

Supplemental Figure 4: Prohibitin is an EM fiducial for studying mitochondrial membrane morphology changes. Back projection of prohibitin particles into raw tomograms identified mitochondria with membrane distortions. (A) A mitochondrial fragment with spherical cristae is juxtaposed by a single tubular crista containing prohibitin (inset). Prohibitin complexes were also identified exposed to the cytosol in mitochondria with outer membrane peels (B and inset). A single layer outer membrane bleb from a mitochondrion is identified by a prohibitin complex on the interior of the bleb (C and inset). A double membrane rupture of a mitochondria is shown with prohibitin present in the cristae below the rupture site (D and inset). (E) Comparison of membrane morphologies from each class (n=85 fragments, 79 with no abnormalities).

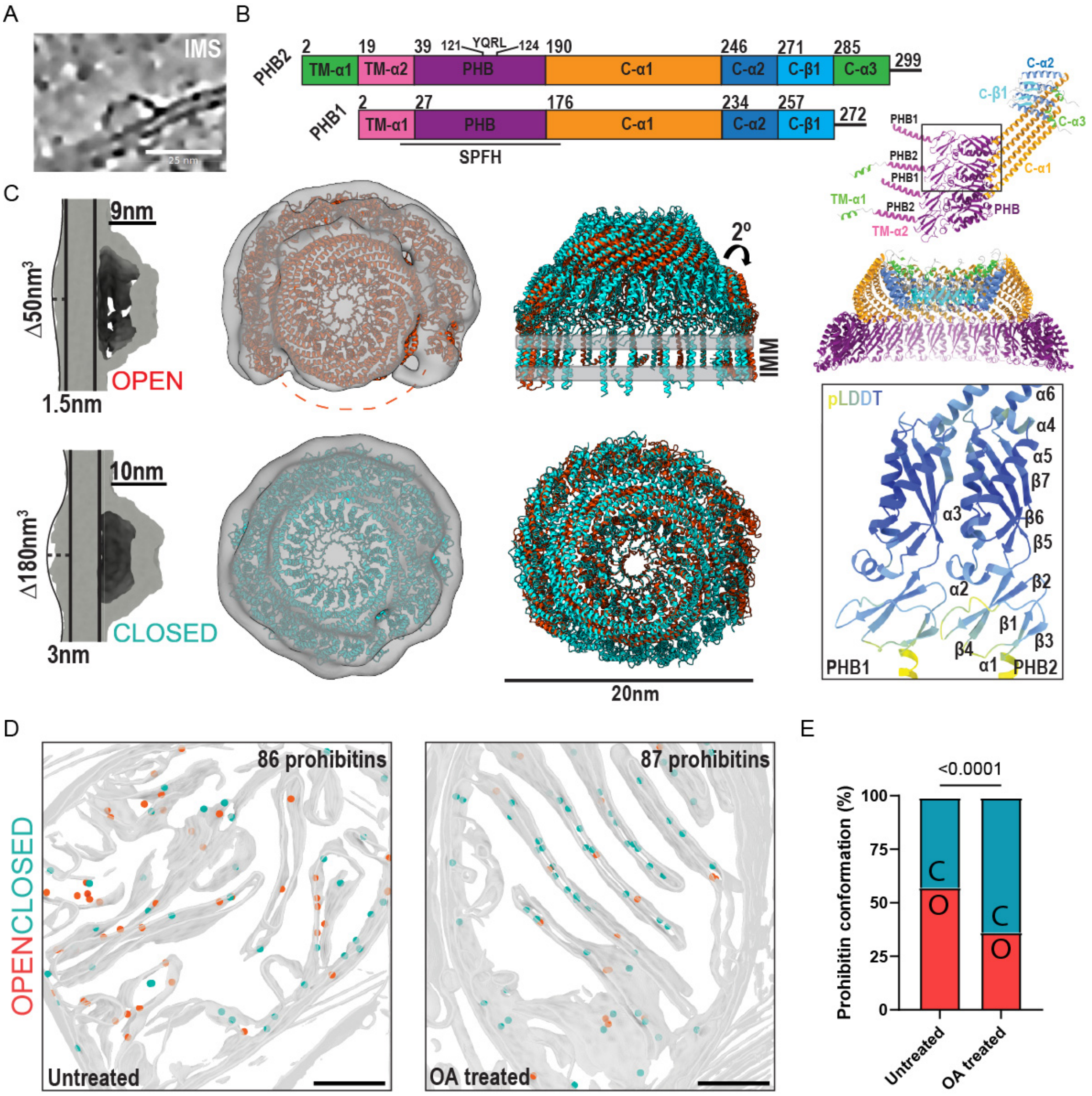

Supplementary Figure 5: Structural comparison of HFLC/K and prohibitin structures. Structures of HFLC/K complex in the open (PDB: 9CZ2) and closed states (PDB: 7VHP) (A). Prohibitin models (open at top, closed at bottom) generated in this study (B). A prohibitin model generated from C11 symmetric processing (PDB: 8RRH) (C). Closed model from this study docked into the density map of putative prohibitin from Chlamydomonas (EMDB-50212) (D).

Movie S1: Representative tomogram of an untreated mitochondria.

Movie S2: Representative tomogram of an OA treated mitochondria

Movie S3: Tomogram of a phagophore with putative BLTPs targeting a damaged mitochondrial fragment.

Movie S4: Tomogram showing membranes enveloping an OA treated mitochondrial fragment.

Movie S5: Morph of prohibitin maps and models illustrating differences between the two conformations.
